# [^18^F]Fluciclovine PET/CT versus [^18^F]DCFPyL PET/CT in patients with biochemical recurrence of prostate cancer after robot-assisted radical prostatectomy: a prospective, single-center, single-arm, comparative imaging trial

**DOI:** 10.1007/s00259-026-07816-3

**Published:** 2026-02-28

**Authors:** Wietske I. Luining, André N. Vis, Maarten L. Donswijk, Jose C. C. Koppes, Pim J. van Leeuwen, Frederik Oudshoorn, Matthijs C. F. Cysouw, Daniela E. Oprea-Lager

**Affiliations:** 1 Department of Urology, Amsterdam University Medical Center, VU university, location VUmc, De Boelelaan 1117, 1081 HV Amsterdam, The Netherlands; 2 Department of Radiology & Nuclear Medicine, Amsterdam University Medical Center, Amsterdam, The Netherlands; 3 Prostate Cancer Network the Netherlands, Amsterdam, The Netherlands; 4 Cancer Center Amsterdam, Amsterdam University Medical Center, Amsterdam, The Netherlands; 5 Department of Nuclear Medicine, Netherlands Cancer Institute-Antoni van Leeuwenhoek, Amsterdam, The Netherlands; 6 Department of Urology, Netherlands Cancer Institute-Antoni van Leeuwenhoek, Amsterdam, The Netherlands; 7 Department of Urology, Spaarne Gasthuis, Haarlem, The Netherlands; 8 Department of Medical Imaging, Radboud University Medical Center, Nijmegen, The Netherlands

**Keywords:** Prostate cancer, Biochemical recurrence, [^18^F]Fluciclovine, FACBC, [^18^F]DCFPyL, PET/CT

## Abstract

**Purpose:**

The current EAU-EANM-ESTRO-ESUR-ISUP-SIOG Guidelines recommend radiolabeled prostate-specific membrane antigen (PSMA), choline, or fluciclovine PET/CT at biochemical recurrence (BCR) of prostate cancer (PCa) after radical prostatectomy. While studies compared [^68^Ga]Ga-PSMA-11 and [^18^F]Fluciclovine, data on ^18^F-labelled PSMA-ligands versus [^18^F]Fluciclovine in patients with low prostate-specific antigen (PSA) levels (< 2 ng/mL) are limited. This study compared the detection rates of [^18^F]Fluciclovine and [^18^F]DCFPyL PET/CT in patients with BCR after robot-assisted radical prostatectomy (RARP). Secondary objectives included stratifying detection rates by PSA level, anatomical regions and assessing inter-observer agreement.

**Methods:**

In this prospective, single-center study, patients with BCR (PSA 0.2-2.0 ng/mL) underwent both [^18^F]Fluciclovine and [^18^F]DCFPyL PET/CT within 15 days. Three blinded nuclear medicine experts independently reviewed all scans. Lesions were classified as positive or negative in predefined regions (i.e., prostate bed, pelvic and extra-pelvic lymph nodes, bone, and visceral). Discrepancies were resolved by consensus, defined as majority agreement (≥ 2/3 readers). Inter-observer agreement was assessed using Fleiss’ kappa.

**Results:**

Overall scan positivity was 44% (22/50 patients) for [^18^F]DCFPyL PET/CT and 24% (12/50) for [^18^F]Fluciclovine PET/CT (p *=* 0.018). Detection rates increased with rising PSA levels for both tracers. Local recurrence was detected by both tracers in 16% (8/50) of patients. Metastatic disease detection rates for [^18^F]DCFPyL versus [^18^F]Fluciclovine were 22% vs. 8.0% for pelvic lymph nodes (p *=* 0.016), and 6.0% versus 2.0% for distant metastases (*p* = 0.50), respectively. Inter-observer agreement was moderate for both tracers (κ = 0.59 for [^18^F]DCFPyL and κ = 0.55 for [^18^F]Fluciclovine).

**Conclusion:**

This study demonstrated the superiority of [^18^F]DCFPyL over [^18^F]Fluciclovine in detecting pelvic lymph node metastases in patients presenting with BCR after RARP at low PSA levels. These findings suggest the potential of [^18^F]DCFPyL PET/CT to facilitate earlier and more personalized salvage treatment strategies.

## Introduction

Prostate cancer (PCa) is the most commonly diagnosed malignancy among men over 50 years in the Western world [1, 2]. The standard treatments for men with localized disease are radical prostatectomy (RP) and local radiation therapy, with or without concomitant androgen deprivation therapy (ADT). Despite these treatments, 27% to 53% of patients experience biochemical recurrence (BCR) of disease [3]. BCR after RP is defined as two consecutive, rising prostate-specific antigen (PSA) levels of at least 0.2 ng/mL. Patients with BCR remain potentially curable. Accurate identification of the site(s) of recurrence is therefore essential to guide the optimal therapeutic approach, including salvage radiation, metastasis-directed therapy (MDT), medical management, or combination strategies [4, 5]. Accurate staging of BCR is therefore crucial for determining prognosis and for selecting and planning potentially curative salvage treatment.

Conventional imaging techniques, such as bone scintigraphy and computed tomography (CT), offer limited sensitivity and specificity for detecting (early) recurrent disease or metastases [6–8]. These methods are outperformed by more sensitive and specific hybrid next-generation imaging modalities (e.g., positron emission tomography/CT (PET/CT)) using recently developed tracers [3]. Currently, the EAU-EANM-ESTRO-ESUR-ISUP-SIOG Guidelines on Prostate cancer recommend the use of radiolabelled prostate-specific membrane antigen (PSMA) ligands, radiolabelled choline or fluciclovine in patients with BCR after radical prostatectomy, if the results will influence subsequent treatment decisions.

[^18^F]Fluciclovine (anti-1-amino-3-¹⁸F-fluorocyclobutane-1-carboxylic acid; FACBC) PET has demonstrated slightly higher accuracy in detecting recurrent disease, compared with [^11^C]Choline PET/CT (38% vs. 32%) [9]. PSMA PET/CT is now increasingly used for both primary staging of newly diagnosed PCa and restaging of recurrent disease. Previous comparative studies in patients with BCR of disease have reported significantly higher detection rates with PSMA tracers than with [^18^F]Fluciclovine. Reported detection rates were 56% versus 26% for [⁶⁸Ga]Ga‑PSMA‑11 and 68% versus 42% for [^18^F]PSMA-1007 [10, 11]. Studies indicate that [^18^F]DCFPyL PET/CT has promising accuracy in localizing recurrent disease [12, 13]. However, the performance of [^18^F]DCFPyL versus [^18^F]Fluciclovine in the same patient cohort, with low serum PSA levels (< 2 ng/mL), has not yet been investigated.

The primary objective of this prospective head-to-head study was to directly compare the per-patient detection rates of [^18^F]Fluciclovine PET/CT and [^18^F]DCFPyL PET/CT in patients with first BCR of disease after robot-assisted radical prostatectomy (RARP). Secondary objectives included evaluation of detection rates stratified by PSA level, across anatomical regions, and assessment of inter-observer agreement.

## Patients and methods

**Fig. 1:**
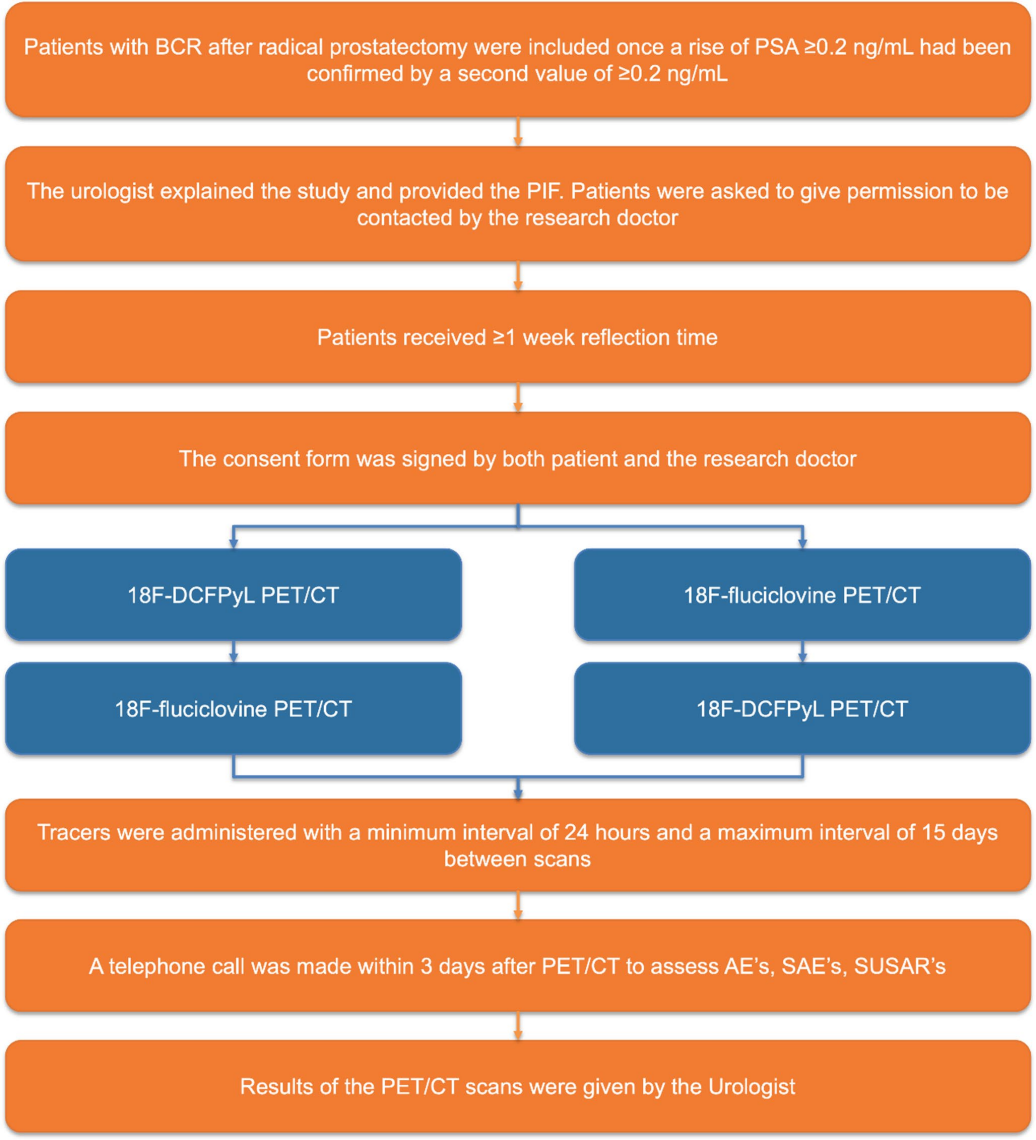
Study chart. BCR = biochemical recurrence; PSA = prostate-specific antigen; PIF = patient information form; PET/CT = positron emission tomography/computed tomography; AE = adverse event; SAE = serious adverse event; SUSAR = suspected unexpected serious adverse reaction

This prospective, single-center, open-label, non-randomized study was conducted at the Amsterdam University Medical Center (UMC). It was designed as a head-to-head comparison of the detection rates of two PET tracers in patients with BCR with low PSA levels (0.2-2.0 ng/mL), following RARP (Fig. 1). The study protocol was approved by the medical ethics committee of the Amsterdam UMC (reference number 2021.0149), and all patients provided written informed consent. Patients were enrolled between January 2022 and April 2024.

### Patient population

Eligible patients were adult men (≥ 18 years) with histologically confirmed PCa who had undergone RARP, with or without an extended pelvic lymph node dissection (ePLND). Only patients with a PSA level between 0.2 and 2.0 ng/mL were included. BCR was defined as a PSA level ≥ 0.2ng/mL on two consecutive measurements or a rising PSA [3]. Key exclusion criteria included histopathological lymph node positivity (pN1) after ePLND, prior salvage therapies, ADT and other concurrent malignancies.

For all patients, comprehensive clinical, biochemical, radiological, and pathological information was collected. Clinical variables included age and clinical tumor stage assessed by digital rectal examination (DRE). Biochemical data comprised PSA at initial diagnosis, most recent PSA at the time of performing PET/CT, and PSA recurrence dynamics (e.g., PSA nadir, PSA level at recurrence, PSA doubling time). Histopathological variables encompassed the percentage of positive biopsy cores (with targeted cores pooled and reported as one positive biopsy core within systematic sets), International Society of Urological Pathology (ISUP) Grade Group after biopsy and RARP [14]. Additional variables included histopathological TNM stage post-surgery and surgical margin status. Imaging data comprised PET/CT specifics, including date, radiopharmaceutical, injected dose (MBq), scan trajectory, reconstruction parameters and imaging findings. Risk stratification was performed according to the European Association of Urology (EAU) classification system [3].

Adverse events (AEs), serious adverse events (SAEs), and suspected unexpected serious adverse reactions (SUSARs) were monitored and recorded according to the protocol-defined criteria.

### Imaging procedure

All patients underwent both [^18^F]Fluciclovine and [^18^F]DCFPyL PET/CT scans using standardized protocols. The interval between scans ranged from a minimum of 24 h to a maximum of 15 days. Scans were performed and interpreted in a non-fixed order and the identity of the administered tracer was not blinded due to the open-label design.

For [^18^F]Fluciclovine, patients fasted for 4 h prior to tracer administration. The [^18^F]Fluciclovine tracer was produced and delivered by GE Healthcare. A standard activity of 370 MBq (± 10%) was injected intravenously while the patient was positioned on the PET/CT scanner bed. PET imaging commenced 3–5 min post-injection (target 4 min). Acquisition was performed from mid-thigh to skull base at 3 min per bed position, resulting in a total scan time of approximately 24 min. Scans were performed in accordance with the procedure guidelines [15].

[^18^F]DCFPyL was synthetized via direct radiofluorination at an on-site cyclotron facility. Whole-body PET was performed 120 min (± 15 min) after intravenous injection of 330 MBq [^18^F]DCFPyL. Imaging was acquired from mid-thigh to the base of the skull at 4 min per bed position. The total acquisition time averaged 30 min. No fasting was required and no diuretics were administered prior to imaging. Scans were conducted in line with the imaging guidelines for PSMA PET/CT [16].

For both tracers, PET was performed using an European Association of Nuclear medicine Research LTd (EARL) calibrated hybrid Philips Ingenuity TF scanner and combined with a low-dose CT (30–120 mAs; 120 kV). All images were corrected for decay, scatter, random coincidences and photon attenuation. Images were reconstructed using the EARL specifications on the quantitative reads of clinical PET/CT studies [17].

### Image evaluation

A nuclear medicine physician initially reviewed both PET/CT scans on-site, without blinding to clinical data. Therapeutic decisions were based on the combined clinical and imaging findings. These decisions were typically made in consultation with a multidisciplinary team consisting of urologists, radiation oncologists, nuclear medicine physicians and medical oncologists. Scan interpretation was in line with the PROMISE criteria [18]. A lesion was considered positive when focal uptake was visually higher than surrounding background activity and not attributable to physiological uptake or benign processes. Supportive anatomical substrate on low-dose CT was used when applicable. No minimum lymph node size threshold was applied and small nodes were scored as positive if uptake was clearly focal and exceeded local background. Equivocal findings were scored as negative.

For study analysis, all PET/CT scans were anonymized and independently evaluated by three nuclear medicine physicians. Readers were blinded to clinical information and to the results of the other scan. A mix of [^18^F]DCFPyL and [^18^F]Fluciclovine scans was presented to the readers in random order. Scans from the same patient were not read consecutively. Each reader assessed the presence of PCa in predefined anatomical regions, including the prostate bed (miTr), pelvic lymph nodes (miN1), extra-pelvic lymph nodes (miM1a), bone lesions (miM1b), and other organs (miM1c). These sites were scored as positive or negative for suspected BCR. Discrepancies between the three readers were resolved by consensus, defined as majority agreement (≥ 2 of 3 readers). Only the blinded independent readings were used for the analysis. The original on-site interpretations, conducted with knowledge of all clinical information, were not incorporated or analyzed for the study outcomes.

### Outcomes

The primary outcome was to compare the per-patient detection rates of [^18^F]Fluciclovine and [^18^F]DCFPyL PET/CT, independent of follow-up data. Secondary outcomes included detection rates across anatomical regions, detection rates stratified by PSA level (i.e., 0.2–0.5, 0.51–1.0, and 1.01–2.0 ng/mL), and the assessment of inter-observer agreement.

### Statistical analysis

Sample size was calculated using a one-sided McNemar test (α = 0.05, power = 80%), requiring 50 patients to detect a 30% difference in detection rates between tracers. Baseline characteristics were summarized descriptively. Detection rates were reported on a per-patient basis (≥ 1 PET-positive anatomical region) and per-region basis (i.e., prostate bed, pelvic lymph nodes, extra-pelvic lymph nodes, bone, visceral). Positivity was defined by PET-positive findings regardless of pathology or imaging/clinical follow-up. Paired comparisons between tracers were performed using McNemar’s test. To control for multiplicity, hypothesis testing was limited to the primary and region-specific endpoints.

Detection rates were also summarized by PSA level at PET/CT. These subgroup analysis were exploratory and are presented descriptively. Inter-observer agreement for each tracer was assessed using Fleiss’ kappa, interpreted according to the Landis and Koch classification: 0.00 (poor), 0.01–0.20 (slight), 0.21–0.40 (fair), 0.41–0.60 (moderate), 0.61–0.80 (substantial), and 0.81–1.00 (almost perfect) reproducibility [19]. Bootstrapped 95% confidence intervals were computed for Fleiss’ kappa statistics to estimate inter-observer agreement uncertainty.

A *p*-value ≤ 0.05 was considered statistically significant. All analyses were performed in RStudio (version 4.4.3).

## Results

### Patient population

The median age of the study population was 72 years (IQR 67–76). At first PET/CT, the median PSA level was 0.31 ng/mL (IQR 0.25–0.40). Baseline clinical, biochemical, and pathological characteristics are summarized in Table 1. All patients underwent both [¹⁸F]Fluciclovine and [¹⁸F]DCFPyL PET/CT scans in random order with a median interval of 4 days (IQR 3–7). The median injected activities were 384 MBq (IQR 363–397) and 335 MBq (IQR 322–346) for [¹⁸F]Fluciclovine and [¹⁸F]DCFPyL. No serious tracer-related adverse events were observed.

**Table 1:** Patient characteristics of all included patients

Characteristics		All patients
*n*		50
Age at first PET/CT, median (IQR)		72 [67, 76]
PSA at initial diagnosis (ng/mL), median (IQR)		8.20 [6.40, 12.25]
Clinical tumor stage, no. (%)	cT1	24 (48.0)
	cT2a/b	23 (46.0)
	cT2c	2 (4.0)
	cT3	1 (2.0)
Highest grade group according to ISUP, no. (%)	ISUP 1/2	29 (58.0)
	ISUP 3	10 (20.0)
	ISUP 4	7 (14.0)
	ISUP 5	4 ( 8.0)
Percentage of positive biopsies, median (IQR)*		50.00 [28.57, 62.50]
Risk classification according to the EAU, no. (%)	Low risk	2 ( 4.0)
	Favorable intermediate risk	15 (30.0)
	Unfavorable intermediate risk	16 (32.0)
	High risk	17 (34.0)
RARP T stage, no. (%)	pT1a–c	1 ( 2.0)
	pT2a–c	22 (44.0)
	pT3a	14 (28.0)
	pT3b	13 (26.0)
RARP grade group according to ISUP, no. (%)	ISUP 1	1 ( 2.0)
	ISUP 2	19 (38.0)
	ISUP 3	19 (38.0)
	ISUP 4	3 ( 6.0)
	ISUP 5	8 (16.0)
Surgical margin status, no. (%)	Negative	25 (50.0)
	Positive	25 (50.0)
RARP N stage, no. (%)	Negative**	26 (52.0)
	ePLND not performed	24 (48.0)
Biochemical recurrence, no. (%)	BCR	42 (84.0)
	BCP	8 (16.0)
Risk group biochemical recurrence according to the EAU, no. (%)	Low risk	16 (32.0)
	High risk	34 (68.0)
PSA doubling time (months), median (IQR)	=<12	27 (54.0)
	> 12	23 (46.0)
PSA at first PET/CT (ng/mL), median (IQR)		0.31 [0.25, 0.40]
PSA at PET/CT per subgroup (ng/mL), no. (%)	0.20–0.50	41 (82.0)
	0.51-1.00	7 (14.0)
	1.01-2.00	2 ( 4.0)

PET/CT = Positron Emission Tomography/Computed Tomography; IQR = Interquartile Range; PSA = Prostate-Specific Antigen; ISUP = International Society of Urological Pathology; EAU = European Association of Urology; RARP = Robot-Assisted Radical Prostatectomy; T stage = Tumour Stage; N stage = Nodal Stage; ePLND = extended Pelvic Lymph Node Dissection; BCR = Biochemical Recurrence; BCP = Biochemical Persistence

*Targeted prostate biopsies were pooled and reported as a single core within the systematic biopsies

**Among the 50 patients, 26 underwent ePLND, all of whom had negative lymph nodes (pN0)

### Per-patient detection rates

At the per-patient level, the overall detection rate of PET/CT-positive lesions was 44% (22/50) for [¹⁸F]DCFPyL and 24% (12/50) for [¹⁸F]Fluciclovine PET/CT (p *=* 0.018). Concordance between tracers was observed in 32 of 50 patients (64%), of which 8 (16%) had positive findings and 24 (48%) had negative findings with both tracers. Discordant results occurred in 18 patients (36%), including 14 patients with positive findings on [¹⁸F]DCFPyL but negative results on [¹⁸F]Fluciclovine PET/CT, and 4 patients with the reverse pattern (Table 2).

**Table 2:** Contingency table of the overall and region-specific detection rates for [^18^F]DCFPyL and [^18^F]Fluciclovine PET/CT

	Overall^§^		miTr*		miN*		miM*	
	F−	F+	F−	F+	F−	F+	F−	F+
D–	24	4	37	5	39	0	47	0
D+	14	8	5	3	7	4	2	1
p-value	0.031		1.0		0.016		0.50	

D– = DCFPyL-negative; D + = DCFPyL-positive; F– = Fluciclovine-negative; F + = Fluciclovine-positive; mi = molecular imaging;

Tr = local tumor recurrence; N = pelvic lymph node metastases; M = distant metastases

^**§**^The overall detection rate refers to the proportion of patients with at least one PET/CT-positive anatomical region

*Values represent counts of regions with or without uptake per tracer

### Per-region detection rates

**Fig. 2:**
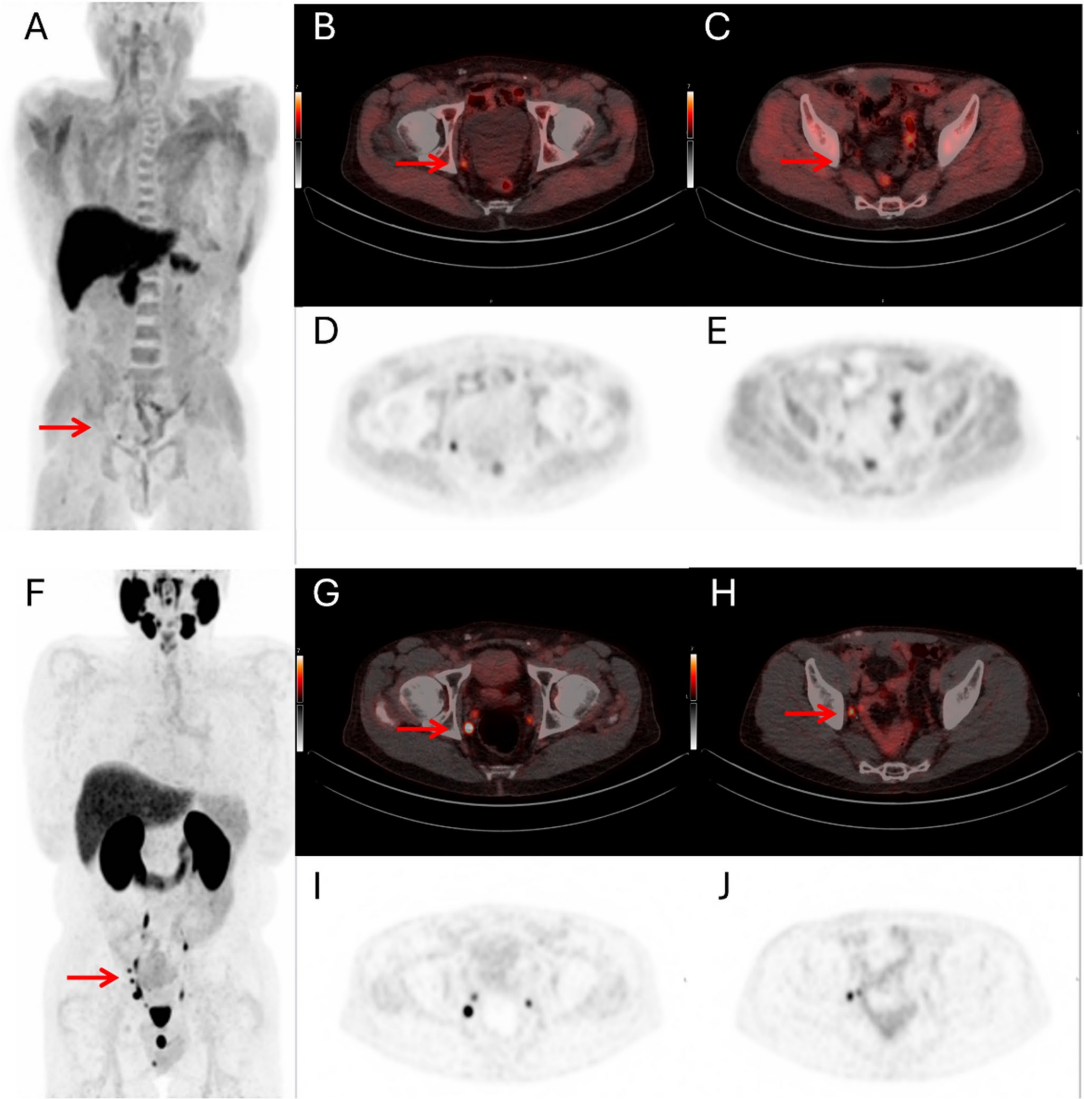
Clinical example of a 73-year-old patient with biochemical recurrence of prostate cancer after radical prostatectomy with extended pelvic lymph node dissection (ISUP Grade Group 3, pT2cN0, negative surgical margins). The PSA level at the time of PET/CT was 0.21 ng/mL. [^18^F]Fluciclovine PET/CT was performed first, followed 7 days later by [^18^F]DCFPyL PET/CT. Both [^18^F]Fluciclovine and [^18^F]DCFPyL PET/CT showed no abnormal tracer uptake in the prostate bed, indicating no evidence of local recurrence. Maximum Intensity Projection (MIP) images, axial fused PET/CT images and axial PET images of [^18^F]Fluciclovine (**A-E**) and [^18^F]DCFPyL (**F-J**). [^18^F]Fluciclovine PET shows only a solitary, moderately avid right obturator lymph node (7 mm) (**B**,** D**). The same lymph node is visible on [^18^F]DCFPyL PET (7 mm; SUVmax 29) (**G**,** I**). In contrast, [^18^F]DCFPyL PET/CT demonstrates multiple additional lymph nodes with increased PSMA expression (**F**), including a right iliac node (3 mm) detected only on [^18^F]DCFPyL (**H**,** J**) and not on [^18^F]Fluciclovine PET (**C**,** E**). Additional other small nodes include a mid-obturator node (3.5 mm) and two mesorectal nodes (< 3 mm), which are not shown in this figure. Lesions are highlighted with red arrows

On anatomical analysis, local recurrence was detected in 8 out of 50 patients (16%) by both [¹⁸F]DCFPyL and [¹⁸F]Fluciclovine PET/CT, including 3 concordant positives (both tracers), with balanced discordants (5 per tracer). [¹⁸F]DCFPyL PET/CT identified pelvic lymph node metastases in 11 patients (22%) and distant metastatic disease in 3 patients (6.0%). The corresponding detection rates for [¹⁸F]Fluciclovine were 8.0% and 2.0%, respectively (Table 3; Fig. 2). For [¹⁸F]DCFPyL, the distant metastases involved two patients with extra-pelvic nodal disease (miM1a) and one patient with suspected miM1c disease. No bone metastases were observed. For [¹⁸F]Fluciclovine, one patient had an extra-pelvic nodal metastasis corresponding to one of the miM1a cases also identified with [¹⁸F]DCFPyL. No skeletal or visceral metastases were observed. A statistically significant difference between the tracers was observed in the detection of pelvic lymph nodes (*p* = 0.016). No significant differences were observed in the detection of local recurrence or distant metastases.

Discordant results for local tumor recurrence were evenly distributed, with five patients positive on [¹⁸F]DCFPyL only and five patients positive on [¹⁸F]Fluciclovine only. For pelvic lymph nodes and distant metastases, all discordant cases were detected on [¹⁸F]DCFPyL only (7 vs. 0 and 2 vs. 0 patients, respectively). These findings are detailed in Table 2.

**Table 3:** Detection rate per-anatomical region

Region	[¹⁸F]DCFPyL	[¹⁸F]Fluciclovine
miTr	8/50 (16.0%)	8/50 (16.0%)
miN	11/50 (22.0%)	4/50 (8.0%)
miM	3/50 (6.0%)	1/50 (2.0%)

mi = molecular imaging; Tr = local tumor recurrence; N = pelvic lymph node metastases; M = distant metastases

### PSA-stratified detection rates

Detection rates increased progressively with higher PSA levels for both tracers. For [¹⁸F]DCFPyL, the detection rates were 39%, 57%, and 100%, and for [¹⁸F]Fluciclovine, these rates were 17%, 43%, and 100% for the 0.2–0.5, 0.51–1.0, and 1.01–2.0 ng/mL PSA subgroups, respectively (Table 4).

**Table 4:** Per-patient detection rates stratified by PSA level at first PET/CT

PSA subgroups (ng/mL)	[^18^F]DCFPyL	[^18^F]Fluciclovine
0.20–0.50	16/41 (39%)	7/41 (17%)
0.51-1.00	4/7 (57%)	3/7 (43%)
1.01-2.00	2/2 (100%)	2/2 (100%)

PSA = Prostate-Specific Antigen; PET/CT = Positron Emission Tomography/Computed Tomography

### Inter-observer variability

Inter-reader agreement was moderate on the patient level, with Fleiss’ kappa values of 0.59 for [¹⁸F]DCFPyL and 0.55 for [¹⁸F]Fluciclovine. Subgroup analysis per anatomical region showed substantial agreement for local recurrence (κ = 0.72 and 0.68), substantial agreement for pelvic lymph nodes with [¹⁸F]DCFPyL (κ = 0.72), but only fair agreement with [¹⁸F]Fluciclovine (κ = 0.40), and fair agreement for distant metastatic disease with both tracers (κ = 0.28 and 0.23). Detailed estimates, including 95% confidence intervals, are provided in Table 5.

**Table 5:** Inter-reader agreement for [¹⁸F]DCFPyL and [¹⁸F]Fluciclovine PET/CT

Outcome	[¹⁸F]DCFPyL κ (95% CI)	[¹⁸F]Fluciclovine κ (95% CI)
Local recurrence	0.72 (0.47–0.89)	0.68 (0.42–0.86)
Regional lymph nodes	0.72 (0.52–0.88)	0.40 (0.02–0.68)
Distant metastatic disease*	0.28 (0.05–0.41)	0.23 (0.00–0.49)

PET/CT = Positron Emission Tomography/Computed Tomography; κ = Fleiss’ kappa; CI = Confidence Interval

*Distant metastatic disease was defined as the presence of extra-pelvic lymph node, bone, or other soft-tissue lesions

## Discussion

This prospective, head-to-head study is the first to directly compare the ^18^F-labelled PSMA tracer [^18^F]DCFPyL with the previously established reference tracer [^18^F]Fluciclovine in patients with low PSA levels BCR of PCa after RARP. [^18^F]DCFPyL achieved a significantly higher detection rate than [^18^F]Fluciclovine, particularly in the detection of pelvic lymph node metastases. This highlights the potential of [^18^F]DCFPyL PET/CT to determine whether nodal metastatic disease is present and to define its extent. These findings directly inform treatment decisions regarding either potentially curative salvage therapies or systemic treatment.

Conventional imaging modalities such as CT, MRI, and bone scintigraphy have limited sensitivity for detecting recurrence of disease at low PSA levels. Advances in molecular imaging, particularly PET/CT with next-generation radiotracers, have substantially improved diagnostic accuracy in this setting [7, 8]. Reflecting this progress, the European Association of Urology (EAU) currently recommends PSMA PET/CT as the first-line imaging modality for biochemical recurrence, reserving [^18^F]Fluciclovine for cases where PSMA PET/CT is unavailable and PSA levels exceed 1 ng/mL [3].

Our results support and extend findings from previous studies. Loeff et al. demonstrated a similar significant superiority of [^18^F]PSMA-1007 over [^18^F]Fluciclovine (68% vs. 42%; *p* < 0.001), particularly in patients with low PSA levels (61% vs. 25% for PSA 0.2–0.5 ng/mL) [11]. Calais et al. reported higher detection rates for [^68^Ga]Ga-PSMA-11 compared to [^18^F]Fluciclovine (56% vs. 26%; *p* *=* 0.0026) [10]. Collectively, these findings demonstrate that PSMA-targeting tracers, regardless of the isotope used (i.e., ¹⁸F or ⁶⁸Ga), consistently outperform [^18^F]Fluciclovine in localizing recurrence in BCR, especially in early disease stages where curative salvage treatment is considered feasible. While minor differences in the detection rate between PSMA tracers may exist due to pharmacokinetic properties, their diagnostic superiority over [^18^F]Fluciclovine is robust and reproducible [20, 21].

No significant difference was observed in local tumor recurrence detection between [¹⁸F]Fluciclovine and [¹⁸F]DCFPyL, although [¹⁸F]Fluciclovine was expected to be favored due to its hepatobiliary clearance and limited renal excretion in the urinary bladder. However, the renally excreted [¹⁸F]DCFPyL demonstrated similar detection rates for local tumor recurrence and a higher detection rate for pelvic lymph node metastases than [¹⁸F]Fluciclovine. These differences likely reflect tracer-specific pharmacokinetic and biodistribution characteristics, along with preferred dose and acquisition time [22, 23]. Delayed imaging with [¹⁸F]DCFPyL enhances lesion-to-background contrast through increased tumor uptake and background clearance, while its high affinity for PSMA-expressing PCa cells enables detection of metastatic deposits as small as 2 to 3 mm, approaching the spatial resolution limits of current digital PET/CT scanners [22, 24]. In contrast, [¹⁸F]Fluciclovine, a synthetic amino acid PET tracer (Fluor-18; anti-1-amino-3-¹⁸F-fluorocyclobutane-1-carboxylic acid) analogous to L-leucine, is taken up by PCa cells via upregulated amino acid transporters, primarily LAT1 (L-type amino acid transporter 1) and ASCT2 (alanine-serine-cysteine transporter 2) [25]. Early imaging with [¹⁸F]Fluciclovine results in higher physiologic background activity in blood pool, muscle, and bone marrow [23]. This may reduce specificity and sensitivity for local recurrence or small nodal metastases.

Our region-specific analysis demonstrated significantly higher pelvic nodal detection with [^18^F]DCFPyL PET/CT compared with [¹⁸F]Fluciclovine (22% vs. 8%; *p* *=* 0.008). This result is consistent with the findings of Calais et al., who also reported superior pelvic nodal detection using [⁶⁸Ga]Ga-PSMA-11 (30% vs. 8%; p *=* 0.0034) [10]. In contrast, Loeff et al. observed no significant difference between [^18^F]PSMA-1007 and [^18^F]Fluciclovine in pelvic lymph node detection (22% vs. 20%; *p* *=* 1.000) [11]. Regarding distant metastases, we observed low detection rates with both tracers (6% vs. 2%; difference not significant), hampering thorough comparison. This pattern mirrors the results from Loeff et al. (16% vs. 12%), but contrasts with the study by Calais et al., in which [⁶⁸Ga]Ga-PSMA-11 identified a higher proportion of distant metastases (16% vs. 0%; *p* *=* 0.0078). These discrepancies might reflect differences in patient populations. Our cohort consisted exclusively of patients after RARP with very low PSA values (median 0.31 ng/mL; 82% <0.50 ng/mL) and predominantly intermediate-risk disease. The cohort studied by Loeff et al. included only 82% of patients after RALP, but otherwise closely resembled ours (median PSA 0.38 ng/mL; 56% <0.50 ng/mL). In contrast, Calais et al. included a substantially higher proportion of high- and very-high-risk patients according to the National Comprehensive Cancer Network (NCCN) classification (60%). In addition, PSA levels were higher in that cohort (median 0.48 ng/mL; only 26% <0.50 ng/mL), which likely explains the increased rates of nodal and distant metastases.

A key strength of our study is the prospective paired design with blinded independent readings. This approach minimizes interpretive bias and enhances the reliability of the comparative findings. Overall, the moderate inter-reader agreement for both tracers (κ = 0.59 for [^18^F]DCFPyL and κ = 0.55 for [^18^F]Fluciclovine) is consistent with other prospective studies in early BCR of PCa [10, 11]. Regionally, agreement patterns favored PSMA PET/CT for local recurrence and pelvic lymph nodes, whereas reproducibility for distant metastases was limited, most likely due to the low number of positive findings in this early-stage population.

Despite these promising results, this study has inherent limitations. First, histopathological validation of the detected lesions was not performed, nor was imaging or clinical follow-up used to confirm PET-positive findings. This represents a limitation, as the true false-positive rate of [^18^F]DCFPyL cannot be quantified, and our results should therefore be interpreted primarily as a comparison of detection rates rather than definitive diagnostic accuracy. This reflects clinical practice, in which such histopathological confirmation is not always feasible or performed (partly due to the high specificity of the PSMA ligands). This limitation was partially mitigated by independent assessment by three blinded nuclear medicine experts who reviewed all scans. Second, the limited sample size restricted detailed subgroup analyses and may impact the generalizability of some of our findings. Third, patients with pN1 disease were excluded as a distinct prognostic group, which may limit the applicability of our findings to selected patients with undetectable postoperative PSA and no adjuvant treatment. Finally, although early and accurate detection of disease recurrence is expected to improve patient outcomes, prospective studies are required to confirm its impact on long-term survival, quality of life, and treatment decision-making to fully establish the clinical benefit of improved imaging accuracy. Currently, a survival benefit associated with the use of next-generation imaging tracers has not been definitively demonstrated.

## Conclusion

This prospective, open-label head-to-head study compared [^18^F]DCFPyL PET/CT to [^18^F]Fluciclovine PET/CT in patients with biochemical recurrence after radical prostatectomy with low PSA levels (< 2.0 ng/mL). [^18^F]DCFPyL PET/CT demonstrated a significantly higher detection rate, particularly at low PSA levels and for pelvic lymph node metastases. These findings suggest the potential of [^18^F]DCFPyL PET/CT to aid clinical management by facilitating earlier and more accurate localization of recurrent prostate cancer, thereby supporting more personalized salvage treatment strategies.

## Data Availability

The datasets used and/or analyzed during the current study are available from the corresponding author on reasonable request.
